# AUXIN RESPONSE FACTOR3 Regulates Compound Leaf Patterning by Directly Repressing *PALMATE-LIKE PENTAFOLIATA1* Expression in *Medicago truncatula*

**DOI:** 10.3389/fpls.2017.01630

**Published:** 2017-09-20

**Authors:** Jianling Peng, Ana Berbel, Francisco Madueño, Rujin Chen

**Affiliations:** ^1^Noble Research Institute, Ardmore OK, United States; ^2^Insituto de Biología Molecular Celular de Plantas, Consejo Superior de Investigaciones Científicas, Universidad Politécnica de Valencia Valencia, Spain

**Keywords:** auxin signaling, compound leaf development, adaxial-abaxial polarity regulation, *MtARF*3, *Medicago truncatula*

## Abstract

Diverse leaf forms can be seen in nature. In *Medicago truncatula, PALM1* encoding a Cys(2)His(2) transcription factor is a key regulator of compound leaf patterning. PALM1 negatively regulates expression of *SGL1*, a key regulator of lateral leaflet initiation. However, how *PALM1* itself is regulated is not yet known. To answer this question, we used promoter sequence analysis, yeast one-hybrid tests, quantitative transcription activity assays, ChIP-PCR analysis, and phenotypic analyses of overexpression lines and mutant plants. The results show that *M. truncatula* AUXIN RESPONSE FACTOR3 (MtARF3) functions as a direct transcriptional repressor of *PALM1*. MtARF3 physically binds to the *PALM1* promoter sequence in yeast cells. MtARF3 selectively interacts with specific auxin response elements (AuxREs) in the *PALM1* promoter to repress reporter gene expression in tobacco leaves and binds to specific sequences in the *PALM1* promoter *in vivo*. Upregulation of *MtARF3* or removal of both *PHANTASTICA* (*PHAN*) and *ARGONAUTE7* (*AGO7*) pathways resulted in compound leaves with five narrow leaflets arranged in a palmate-like configuration. These results support that MtARF3, in addition as an adaxial-abaxial polarity regulator, functions to restrict spatiotemporal expression of *PALM1*, linking auxin signaling to compound leaf patterning in the legume plant *M. truncatula.*

## Introduction

Leaves are initiated from the flanks of the shoot apical meristem (SAM). The class I Knotted-like homeobox transcription factor genes (*KNOXI*s), which are required to promote and maintain the meristematic activity of SAM, are downregulated at the incipient sites of leaf primordia (Jackson et al., 1994; Lincoln et al., 1994; Long et al., 1996). In simple leaf species, *KNOXI* genes are permanently downregulated (Long et al., 1996). On the other hand, in most eudicot species with compound leaves, including tomato (*Solanum lycopersicum*) and *Cardamine hirsuta, KNOXI* genes are reactivated in developing leaf primordia, and this reactivation is required for compound leaf development (Hareven et al., 1996; Hay and Tsiantis, 2006; Barkoulas et al., 2008; Shani et al., 2009). In the subclade of legumes (Fabaceae), the inverted repeat lacking clade (IRLC), including pea (*Pisum sativum*) and *Medicago* (Wojciechowski et al., 2000), *KNOXI* genes are not reactivated during compound leaf development (Hofer et al., 2001; Champagne et al., 2007; Peng et al., 2011). Instead, *UNIFOLIATA* (*UNI*) and *SINGLE LEAFLET1* (*SGL1*), orthologs of the *Arabidopsis thaliana LEAFY* (*LFY*) gene, are required for the initiation of lateral leaflet primordia (Hofer et al., 1997; Champagne et al., 2007; Wang et al., 2008).

It has been reported that *PALMATE-LIKE PENTAFOLIATA1* (*PALM1*) acts as a key determinant of trifoliate leaves in *Medicago truncatula*. Loss of function mutations of *PALM1* result in palmate-like pentafoliate leaves in contrast to trifoliate leaves in WT plants (Chen et al., 2010). *PALM1* gene encodes a novel Cys(2)His(2) zinc finger transcription factor, which can binds to the *SGL1* promoter sequence and negatively regulates *SGL1* transcription (Chen et al., 2010; Ge et al., 2010).

Plant leaves usually exhibit a flat, bifacial structure with distinct adaxial and abaxial identities (Kidner and Timmermans, 2010; Yamaguchi et al., 2012; Nakata and Okada, 2013; Fukushima and Hasebe, 2014). The adaxial side of the leaf develops tightly packed layers of palisade mesophyll cells, while the abaxial side of the leaf develops loosely packed spongy mesophyll cells and a high density of stomata. The proper specification of the adaxial/abaxial domains in leaf primordia is critical for leaf blade expansion (Emery et al., 2003). *PHANTASTICA* (*PHAN*) was first identified as a key adaxial-abaxial polarity gene in *Antirrhinum majus* (Waites et al., 1998). In *A. majus, phan* mutant produces radialized leaves, in which the adaxial identity was abaxialized (Waites et al., 1998). Interestingly, loss of function mutations in the *PHAN* orthologs, *ASYMMETRIC LEAVES1* (*AS1)* and *ROUGH SHEATH2* (*RS2)* in *A. thaliana* and maize (*Zea mays*), respectively, only have mild effects on the adaxial-abaxial polarity of leaves (Schneeberger et al., 1998; Byrne et al., 2000). In *M. truncatula, phan* mutant displays minor leaf polarity defects especially at a late developmental stage (Ge and Chen, 2014). Independent studies have shown that AUXIN RESPONSE FACTOR3 (ARF3)/ARF4 play redundant roles in determining the leaf abaxial identity (Fahlgren et al., 2006; Garcia et al., 2006; Hunter et al., 2006). In *Arabidopsi*s, single *arf3* mutants display partial loss of the abaxial identity in petals, whereas *arf3;arf4* double mutants display severe polarity defects, including narrow, upwardly curled leaves, and ectopic blade outgrowth on the abaxial side (Pekker et al., 2005). In *Arabidopsis*, both *ARF3* and *ARF4* RNAs are targeted for degradation by the *trans*-acting short-interfering RNA (*ta-siRNA*), *tasiR-ARF*. *tasiR*-*ARF* is derived from the non-coding *TAS3* gene, whose RNA is initially targeted by miR390 for cleavage (Allen et al., 2005). Biogenesis and stabilization of *tasiR-ARF* requires *TAS3*, miR390, and AGONAUTE7 (AGO7), SUPPRESSOR OF GENE SILENCING3 (SGS3), RNA-DEPENDENT RNA POLYMERASE6 (RDR6), and DICER-LIKE4 (DCL4) proteins (Adenot et al., 2006; Fahlgren et al., 2006; Garcia et al., 2006). Loss of function mutations of *ago7, sds3*, or *rdr6* result in reduction of *tasiR-ARF* and over accumulation of *ARF3* and *ARF4* transcripts. Mutant plants exhibit leaf polarity and heteroblastic defects. Genetic studies show that impairment of the *tasiR-ARF* pathway enhances the defects of the *asymmetric leaves1* (*as1*) mutant in leaf adaxial-abaxial partitioning and margin development (Garcia et al., 2006; Xu et al., 2006). AS1 can bind to the *ARF3* promoter and repress *ARF3* transcription in *Arabidopsis* (Iwasaki et al., 2013). Thus, *AS1* and the *tasiR-ARF* pathway negatively regulate *ARF3* expression at the transcriptional and post-transcriptional level, respectively.

It is still not clear whether and how ARF3 regulates leaf patterning in *M. truncatul*a that forms dissected leaves. In this study, we show that *M. truncatula* ARF3 acts as a negative transcriptional repressor of *PALM1* gene expression. We show that ARF3 binds to putative auxin response elements (AuxREs) in the promoter region of *PALM1* and represses expression of reporter gene activities in transcription activity assays in tobacco leaves.

## Materials and Methods

### Plant Materials

*Medicago truncatula phan* (*mtphan*), *palm1* (*palm1-5), mtago7 (mtago7-1)*, and *sgl1 (sgl1-2)* mutants were described previously (Wang et al., 2008; Chen et al., 2010; Zhou et al., 2013; Ge et al., 2014). *Medicago* plants were grown in a greenhouse under the following conditions: 24°C/20°C day/night temperature, 16 h/8 h photoperiod and 150 uE/m^2^/sec light intensity.

### Scanning Electron Microscopy

Shoot apices of 2- to 4-week-old seedlings, or rachis and pulvini were subjected to vacuum infiltration in a fixative solution (3% glutaraldehyde in 25 mM phosphate buffer, pH 7.0) for 1 h and then incubated at 4°C overnight. Plant tissues were further fixed with 1.0% osmium tetroxide in the same phosphate buffer overnight and dehydrated in a graded ethanol series. Scanning electron microscopy (SEM) was performed as described previously (Wang et al., 2008).

### Real-time RT-PCR

Total RNA was prepared using the RNeasy Plant Mini Kit (Qiagen). Residual genomic DNA was removed using a DNA-free Kit (Ambion). cDNA synthesis was performed using SuperScript III reverse transcriptase (Invitrogen), starting with 2 μg of total RNA in a 20-μL reaction mix with oligo(dT)_15_ primer (Promega). Real-time RT-PCR was performed using a 7900HT fast real-time PCR system (Applied Biosystems), as previously described (Peng et al., 2011). SDS2.2.1 software (Applied Biosystems) was used to analyze the melting curve to confirm single amplification. PCR efficiency was estimated by using the LinRegPCR program (Ramakers et al., 2003), and transcript levels were determined in reference to the expression of the *M. truncatula ACTIN* gene Medtr3g095530.

### Yeast One-Hybrid

The Matchmaker Gold System (Clontech) was used in yeast one-hybrid assays. The *PALM1* promoter sequence was PCR-amplified, cloned into the pAbAi vector and integrated into the Y1H Gold genome to create the bait strain, according to the manufacturer’s instructions. Coding sequences of *MtARF3* were PCR-amplified from cDNA templates, cloned into the pGADT7 vector and introduced into the bait strain Y1H Gold/*PALM* promoter. Positive clones grown on double selection media (SD-Ura-Leu) were tested on SD-Ura-Leu plates supplemented with Aureobasidin A (100 ng/ml). Primers are listed in Supplementary Table S1. Yeast transformation was performed as previously described (Peng et al., 2011).

### Constructs and Plant Transformation

*MtARF3* was generated by RT-PCR from total RNA extracted from vegetative shoot buds of *M. truncatula*. MtARF3^m^ with mutations in *tasiR*-ARF recognition sites was created by overlapping PCR, cloned into the pENTR/D vector and then transferred into the pEARLEYGATE 202 vector using Gateway cloning technology. *M. truncatula* transformation was performed as previously described (Peng et al., 2011).

### Transcription Activity Assays

Transcription activity assays were carried out essentially as described previously (Hellens et al., 2005), with the following modifications. E2, three times tandem repeats of the *cis*-element TGTCAA flanked by SpeI and NotI recognition sequences, was inserted into the 35S promoter at the -46 nucleotide position by overlapping PCR; the resulting 35S-E2 was cloned into pGREENII 0800-LUC vector between BamHI and NcoI restriction sites to create the 35S-E2::LUC construct. Three tandem repeats of other elements, E1, E3–E10 (Supplementary Figure S1), flanked by SpeI and NotI sequences, were synthesized, annealed, restriction-enzyme-digested and cloned into SpeI- and NotI-digested 35S-E2::LUC vector to generate the 35S-E::LUC reporter constructs. MtARF3^m^ was cloned into the pMDC32 vector as the effector construct. Both the effector and reporter constructs were introduced into the *Agrobacterium tumefaciens* GV3101 strain. The reporter strains were infiltrated into *Nicotiana benthamiana* leaves, together with the control strain, which harbors *GUS* or the effector strain, which harbors *MtARF3^m^*. Firefly luciferase and *Renillia* luciferase were assayed using the dual luciferase assay reagents (Promega, Madison, WI, United States). Briefly, after inoculation and a transient incubation for 2 days, 1 cm leaf disks were harvested and ground in 100 μl of Passive Lysis Buffer. 5 μl of this crude extract was assayed in 100 μl of Luciferase Assay Buffer, and the first chemiluminescence was measured for firefly luciferase activities. 100 μl of Stop and Glow^TM^ buffer was then added, and a second chemiluminescence measurement was made for *Renillia* luciferase activities. Absolute RLU was measured in a GloMax^®^ 96 Microplate Luminometer with Dual Injectors, with 5-s delay and 15-s measurement. Data were collected as ratios of LUC/REN.

### Phylogenetic Analysis

AUXIN RESPONSE FACTOR (ARF) protein sequences in *A. thaliana* from TAIR10 (The *Arabidopsis* Information Resource) were used to identify ARF protein sequences from *M. truncatula* genome project v4.0^1^. Multiple sequences were aligned using ClustalX (Thompson et al., 1994). Phylogenetic trees were reconstructed using the maximum likelihood algorithm implemented in MEGA10^2^, with 1,000 bootstrap replications (Tamura et al., 2007).

### RNA *In Situ* Hybridization

RNA *in situ* hybridization was performed as described previously (Ferrandiz et al., 2000) with minor modifications. The *MtAGO7* probes correspond to a 517-bp sequence of the *MtAGO7* coding sequence. The *MtARF3* probes correspond to a 511-bp sequence of the *MtARF3* coding sequence. Eight-micrometer sections from shoot apices of 2- to 4-week-old seedlings were processed and hybridized with digoxigenin-labeled sense and antisense probes.

### ChIP-PCR Analysis

ChIP was performed using FLAG-MtARF3^m^-overexpressing *Medicago* plants. ChIP-PCR analysis was done as previously described (Kaufmann et al., 2010; Zhu et al., 2012) with some modifications. Plant tissues were pulverized in liquid nitrogen. Powdered tissues were cross-linked in 10 mM Tris-HCl (pH 8.0) containing 1% formaldehyde, supplemented with 0.4 M sucrose, 5 mM β-mercaptoethanol (β-ME) and a complete proteinase inhibitor cocktail (Roche; PI tablets). Cross linking was done for 10 min at room temperature, and the reaction was quenched by the addition of glycine to a final concentration of 125 mM. The slurry was filtered through a mesh (55 μm) and precipitated by centrifugation at 4,000 *g* for 20 min; the pellet was then resuspended in extraction buffer 2 (0.25 M Sucrose, 10 mM Tris-Cl, pH 8.0, 10 mM MgCl_2_, 1% Triton X-100, 5 mM β-ME and the PI cocktail). This was followed by centrifugation at 14,000 *g* for 20 min. The pellet was resuspended in extraction buffer 3 (1.7 M Sucrose, 10 mM Tris-Cl, pH 8, 2 mM MgCl_2_, 0.15% Triton X-100, 5 mM β-ME and the proteinase inhibitor cocktail) and centrifuged at 20,000 *g* for 40 min. The resulting pellet was resuspended in nuclei lysis buffer (10 mM Tris-HCl, pH 8.0, 20 mM EDTA, 400 mM NaCl, 1% Triton X-100 and the PI cocktail). Sonication, 6 X 15 pulses with a 1 min break between each 15 pulses, was used to shear the DNA. Cell debris was removed by centrifugation. ChIP was performed by incubation with rabbit anti-Flag polyclonal antibody (Abcam) and followed by incubation with Protein A/G magnetic beads (Pierce). The beads were washed twice with high-salt wash buffer (500 mM NaCl, 0.2% SDS, 0.5% Triton X-100, 20 mM Tris-HCl, pH 8.0, 2 mM EDTA and the PI tablet), and the chromatin was eluted in the elution buffer (1% SDS and 0.1 M NaHCO_3_) and de-cross-linked at 65°C for 16 h. Cellular RNA and protein were then removed by RNase (Qiagen) and proteinase-K (Applied Biosystems) treatments, respectively. DNA was purified through a purification column. Enrichment of DNA targets in each sample was determined by PCR using primers listed in Supplementary Table S1.

### GenBank Accession Numbers

HM038482, *PALM1*; AY928184, *SGL1*; XM_003593616, *MtARF3*; DQ468322, *MtPHAN*; XM_003613868, *MtAGO7*; JF929904, *MtNAM*; XM_003602497, *ACTIN.*

## Results

### *Medicago truncatula* ARF3 Negatively Regulates *PALM1* Expression

**FIGURE 1:**
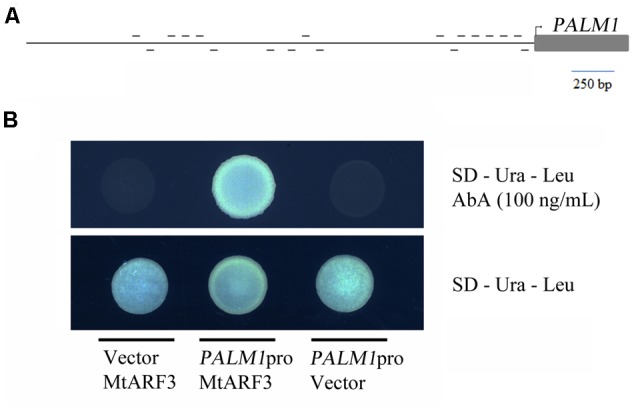
Yeast one-hybrid assays of interactions between MtARF3 and the *PALM1* promoter sequence. **(A)** A schematic drawing of the promoter and coding regions of *PALM1.* Short horizontal lines represent putative auxin response elements (AuxREs; TGTCxx) on the plus (above) and minus (beneath) strands of the promoter sequence. **(B)** Yeast one-hybrid assays, showing interactions between MtARF3 and the *PALM1* promoter sequence. SD, synthetic drop-out media; Ura, uridine; Leu, leucine; AbA, aureobasidin A.

To identify potential regulators of *PALM1* gene expression, we first analyzed whether any specific *cis*-elements are present within the promoter sequence of the *PALM1* gene. This led to the discovery of 18 TGTCXX elements within the 2.5-kb promoter region of *PALM* (Supplementary Figure S1A and **Figure 1A**) (Li et al., 1994; Ulmasov et al., 1995, 1997a). These TGTCXX elements, representing 10 distinct groups (Supplementary Figure S1B), resemble the canonical auxin response element (AuxRE), TGTCTC, which interact with AUXIN RESPONSE FACTORs (ARFs) (Li et al., 1994; Ulmasov et al., 1995, 1997a). Next, we tested whether *M. truncatula* ARF3 (MtARF3) can interact with the *PALM1* promoter sequence in the yeast one-hybrid system. The results show that MtARF3 interacted strongly with the *PALM1* promoter sequence in yeast one-hybrid assays (**Figure 1B**).

**FIGURE 2:**
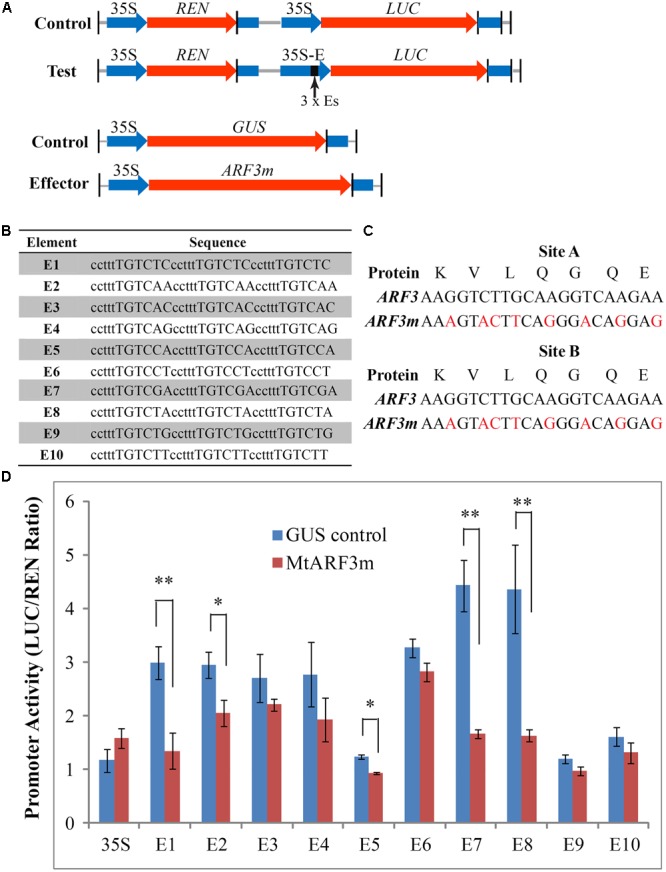
Transcription activity assays in tobacco leaves. **(A)** Schematic drawings of reporter and effector constructs used in quantitative luciferase activity assays in tobacco leaves. **(B)** Putative AuxRE sequences in the *PALM1* promoter region were tested. Capital letters denote the *cis*-elements and lower case letters denote the spacer between *cis*-element repeats. **(C)** Construction of *tasiR*-*ARF*-resistant *MtARF3^m^*. Red letters denote synonymous mutations to disrupt *tasiR*-*ARF* recognition sites A and B. **(D)** Luciferase activity assays in tobacco leaves. Firefly luciferase (LUC) activities were normalized against *Renillia* luciferase (REN) activities. Shown are means ± SD; *n* > 6. Student’s *t*-test, ^∗^*p* < 0.05; Student’s *t*-test, ^∗∗^*p* < 0.01.

It is notable that the canonical AuxRE element (TGTCTC) is present at the -2129 position and a majority of TGTCXX elements are present in the proximity of the translation initiation codon (ATG) of *PALM1* (Supplementary Figure S1). To test whether MtARF3 interacts with the TGTCXX elements in the *PALM1* promoter *in vivo*, we first used quantitative luciferase (LUC) reporter activity assays in tobacco leaves (Hellens et al., 2005). For this, all 10 potential AuxRE elements in the *PALM1* promoter were cloned as three tandem repeats, with the ‘ccttt’ spacer, which was used in the construction of the DR5 auxin response reporter (Ulmasov et al., 1997b), between repeats into the 35S promoter (designated as 35S-Es::LUC; **Figures 2A,B**). Because *ARF3* is post-transcriptionally regulated by *trans*-acting short interfering RNA (*tasiR-ARF*) (Hunter et al., 2006; Montgomery et al., 2008), we generated a modified *MtARF3* (*Mt*ARF3^m^) by mutating the *tasiR-ARF* targeting sites without changing the amino acid sequence (**Figure 2C**). To assess transcription repressor activities of the effector protein, we compared 35S-Es::LUC activities in the presence of the MtARF3^m^ effector protein and the unrelated β-glucuronidase (GUS) protein (**Figure 2D**). The results show that (1) 35S::LUC activities were not significantly different; (2) several potential AuxRE elements, i.e., E3, E4, E9 and E10, exhibited moderate but not significant inhibitory effects on the luciferase activities; and (3) other AuxRE elements, namely E1, E2, E5, E7 and E8, significantly reduced the luciferase activities in the presence of the MtARF3^m^ effector protein compared with that in the presence of the unrelated GUS protein control (**Figure 2D**). These results indicate that MtARF3 can recognize several putative AuxREs that are present in the *PALM1* promoter and function as a transcription repressor in the transcription activity assay in tobacco.

**FIGURE 3:**
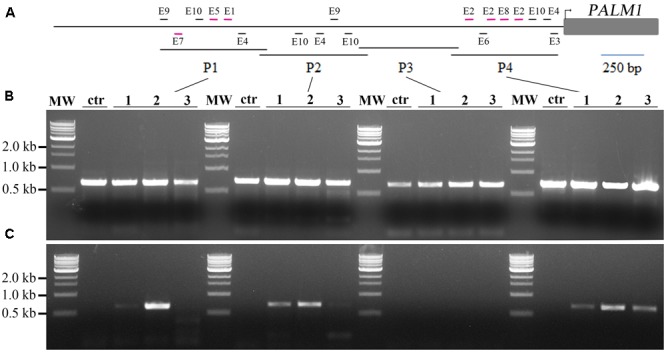
Chromatin immunoprecipitation (ChIP)-PCR assays of interactions between MtARF3 and the *PALM1* promoter *in vivo*. **(A)** A schematic drawing of the *PALM1* promoter, showing location of putative auxin response elements (AuxREs; short horizontal lines) and the promoter fragments, P1–P4. **(B)** PCR analysis shows equal inputs of the DNA samples. **(C)** ChIP-PCR analysis shows enrichment of the *PALM1* promoter fragments, P1, P2 and P4, but not P3, in FLAG-MtARF3-bound chromatins. Lanes MW, 1 kb marker from NEB; ctr, negative control in which wild type plants were used; and 1–3, three independent 35S::*FLAG*-*MtARF3^m^* plants. P1, P2, and P4 each contain several putative AuxREs (short horizontal lines), whereas P3 does not contain any AuxREs. P1 and P4 contain several putative AuxREs (short horizontal lines in purple) that exhibited significant inhibitory effects on luciferase reporter gene expression in the presence of MtARF3^m^ in transcription activity assays in tobacco. Although P2 contains four putative AuxREs that exhibited moderate but not significant inhibitory effects on reporter gene expression in the tobacco system, ChIP-PCR results showed clearly that they are sufficient to be recognized by ARF3 in *Medicago truncatula* plants.

Next, we tested whether MtARF3 can bind to the *PALM1* promoter in *M. truncatula* plants, using chromatin immunoprecipitation coupled with polymerase chain reaction (ChIP-PCR). For this, we first generated independent transgenic *Medicago* plants that overexpress MtARF3^m^-FLAG fusion proteins. ChIP-PCR analysis shows that P1, P2 and P4 regions, but not the P3 region of the *PALM1* promoter were significantly enriched in MtARF3^m^-FLAG-bound chromatins (**Figures 3A–C**). Consistent with these results, P1, P2 and P4 regions, but not the P3 region, of the *PALM1* promoter contain a number of putative AuxREs (**Figure 3A** and Supplementary Figure S1). Particularly, P1 and P4 contain several putative AuxREs, E1, E2, E5, E7, and E8 that exhibited significant inhibitory effects on luciferase activities in tobacco (**Figures 2D, 3A**). Although three different putative AuxRE elements (E4, E9, and E10) that are present in P2 exhibited moderate but not significant inhibitory effects on luciferase activities in the tobacco system (**Figure 2D**), the ChIP-PCR results clearly show that four copies of these elements in P2 are sufficient to be recognized by MtARF3 *in vivo* (**Figures 3A–C**). These results confirm that *PALM1* is a direct target of ARF3 in *M. truncatula* plants

### Overexpression of *MtARF3^m^* Results in Palmate-Like Pentafoliate Leaves with Radialized Blades

**FIGURE 4:**
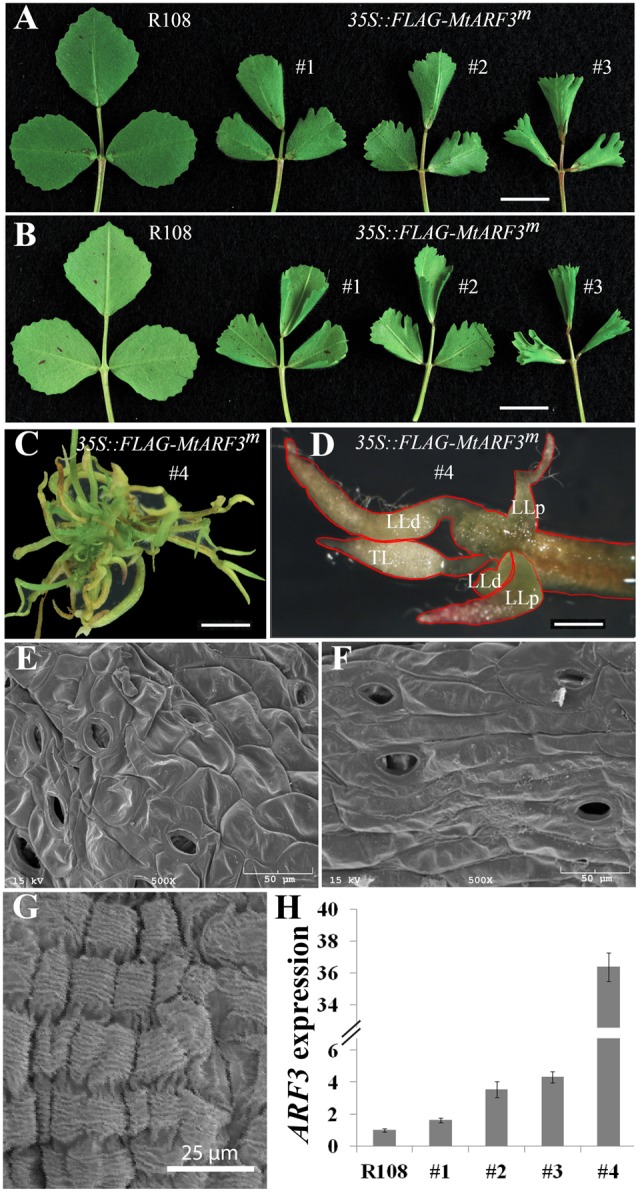
Overexpression of *MtARF3^m^* leads to altered compound leaf patterning in *M. truncatula*. Adaxial **(A)** and abaxial **(B)** views of compound leaves in wild type (R108) and three independent transgenic lines that overexpress *MtARF3^m^* (35S::*FLAG-MtARF3^m^*). Leaflets exhibited deep serration and curling at the distal and proximal margins, respectively, in transgenic plants compared with wild type. **(C,D)** Severe compound leaf phenotypes of a strong *MtARF3^m^* overexpressor. The transgenic plant exhibited pentafoliate leaves with needle-like blades (outlined in red). LLp, proximal lateral leaflets; LLd, distal lateral leaflets; TL, terminal leaflet. **(E,F)** SEM images of the adaxial **(E)** and abaxial **(F)** surface epidermal cells of TL **(D)**. **(G)** A SEM image of epidermal cells of LLd **(D)**. **(H)** Quantitative RT-PCR analysis of *MtARF3* expression in young leaf tissues in *MtARF3^m^* overexpression lines. Shown are means ± SD; *n* = 3. Scale bars: 1 cm **(A,B)**; 2 mm **(C,D)**.

*ARF3* belongs to a large gene family in both *A. thaliana* and *M. truncatula* (Supplementary Figure S2). In *M. truncatula*, many of the *ARF* genes are duplicated, in contrast to the corresponding single genes in *A. thaliana* (Supplementary Figure S2), Because of gene redundancy, loss-of-function or reduced function mutants of single *ARF* genes may not exhibit visible phenotypes in *M. truncatula*. To test whether the interaction between ARF3 and the *PALM1* promoter has physiological relevance, we analyzed transgenic *Medicago* plants, in which *MtARF3*^m^*-FLAG* is ectopically overexpressed by the 35S promoter. Out of four independent lines, three lines exhibited mild leaf phenotypes, including downward curling at the proximal leaf margin and deep serrations at the distal leaf margin, albeit to different degrees (**Figures 4A,B**). These phenotypes are consistent with the role of ARF3 in leaf adaxial-abaxial polarity regulation in *Arabidopsis* and resemble the phenotype of *mtphan* and *mtago7* mutants (Zhou et al., 2013; Ge and Chen, 2014; Ge et al., 2014). Significantly, a strong *MtARF3^m^* overexpression line exhibited severe leaf phenotypes, including some compound leaves with five radialized leaflets arranged in a palmate-like configuration (**Figures 4C,D**). These results suggest that a high level of *MtARF3* overexpression results in proliferation of compound leaves that resemble palmate-like pentafoliate leaves of the *palm1* mutant (Chen et al., 2010), albeit with a severe adaxial-abaxial polarity defect.

Scanning electron microscopy analysis showed that the needle-like structures in the strong *MtARF3^m^* overexpression line are leaflets, because (1) distinct epidermal cell shapes are present on the adaxial and abaxial surface of each needle-like structure (**Figures 4E,F**); (2) each needle-like structure has stomata and trichomes on its surface (**Figures 4E,F**); and (3) each needle-like structure is connected to a petiole or rachis through the motor organ, pulvinus (Chen et al., 2012) (**Figure 4G**).

Reverse transcription (RT)-PCR results show that *MtARF3* transcript level was elevated in all overexpression lines compared with wild type plants (**Figure 4H**), and the severity of the leaf phenotype was correlated with the expression level of *MtARF3* (**Figure 4H**). In the strong *MtARF3^m^* overexpression line, the *MtARF3* transcript level was upregulated about 36 fold over the wild type level, supporting that a high level of *MtARF3* expression is associated with the palmate-like pentafoliate leaf phenotype (**Figure 4H**).

### *mtphan;mtago7* Double Mutant Plants Exhibit Palmate-Like Pentafoliate Leaves

**FIGURE 5:**
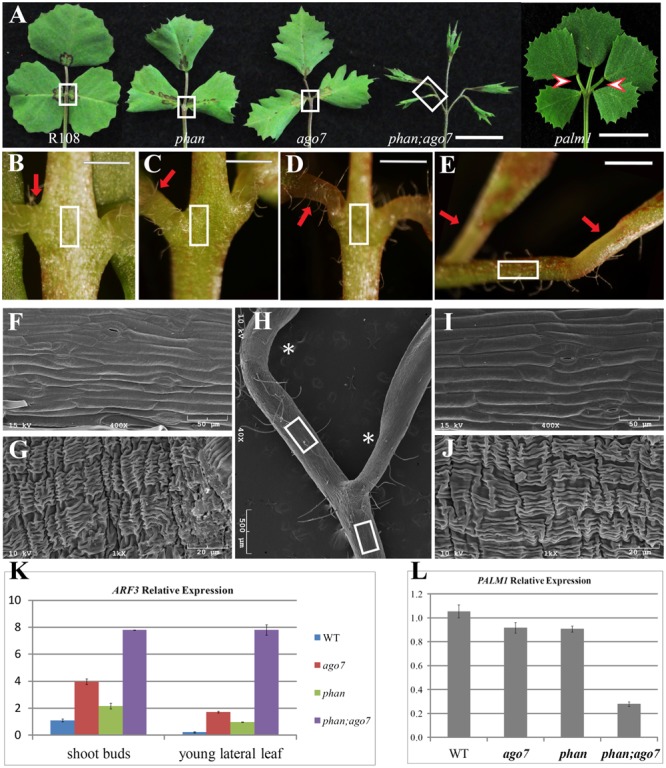
Altered compound leaf patterning in *mtphan;mtago7* double mutant. **(A)** The adaxial view of mature leaves in wild type (R108), and *mtphan, mtago7, mtphan;mtago7* and *palm1* mutants (from left to right). The *mtphan;mtago7* double-mutant leaves were palmate-like pentafoliate with narrow blades and elongated petiolules, in contrast to trifoliate leaves in wild type, and *mtphan* and *mtago7* mutants. Scale bar, 1 cm. Red arrowheads indicate rachises in *palm1* mutants. **(B–E)** Enlarged views of boxed regions in **(A)**, showing the junction between lateral leaflets and petiole in R108 **(B)**, *mtphan* **(C)**, *mtago7* **(D)** and *mtphan;mtago7* **(E)**. Scale bars: 2 mm. **(F)** A representative SEM image of adaxial epidermal cells of the petiole (boxed areas in **B–D**). **(G)** A representative SEM image of epidermal cells of pulvinus (red arrows in **B–D**). **(H)** SEM image of the boxed region from the *mtphan;mtago7* mutant in **(A)**. **(I)** SEM image of epidermal cells of petiolule (boxed regions in **E,H**) in the *mtphan;mtago7* mutant. Note that epidermal cells of petiolule resemble epidermal cells of rachises in WT, and *mtphan* and *mtago7* mutants. **(J)** SEM image of pulvinus (arrows in **E**; asterisks in **H**) in the *mtphan;mtago7* mutant. **(K,L)** Quantitative RT-PCR analysis of expression of *MtARF3* in vegetative shoot buds and young leaves **(K)** and *PALM1* in vegetative shoot buds **(L)** in WT, *mtago7, mtphan*, and *mtphan;mtago7* mutants. Note that the expression level of *PALM1* is negatively correlated with that of *MtARF3*. Shown are means ± SD; *n* = 3.

Since *ARF3* transcript level is regulated both at the transcriptional level by the MYB domain protein, ASYMMETRIC LEAF1 (AS1) (Ikezaki et al., 2010; Kojima et al., 2011; Iwasaki et al., 2013) and at the post-transcriptional level by *tasiR-ARF* (Fahlgren et al., 2006; Montgomery et al., 2008) in *Arabidopsis*, we tested whether *MtARF3* expression can be significantly upregulated in *mtphan;mtago7* double mutants. To generate the *mtphan;mtago7* double mutants, we used *mtphan* and *mtago7* single mutants previously described (Zhou et al., 2013; Ge and Chen, 2014; Ge et al., 2014). Quantitative RT-PCR results show that *MtARF3* transcripts accumulated four and two times higher in vegetative shoot buds in *mtago7* and *mtphan* single mutants, respectively, than wild type (**Figure 5K**). And, the *PALM1* transcript level was slightly but significantly reduced in both *mtago7* and *mtphan* mutants compared with wild type (**Figure 5L**). Similar results on *ARF3* gene expression were obtained in developing leaves in *mtphan* and *mtago7* mutants (**Figure 5K**). Quantitative RT-PCR results further show that the *MtARF3* transcript level was approximately eight times that of the wild type level in the *mtago7;mtphan* double mutant (**Figure 5K**). Most significantly, *mtphan;mtago7* double mutants developed palmate-like pentafoliate leaves with narrow blades (**Figure 5A**). The palmate-like pentafoliate leaf phenotype of the double mutant resembles that of loss-of-function *palm1* mutants, although the *palm1* mutant did not exhibit adaxial-abaxial polarity defects (**Figure 5A**). Interestingly, in the R108 background, the petiolules of the ectopic lateral leaflets appear to diverge from secondary rachises in the *mtphan;mtago7* double mutants and to a less degree also in the *palm1* mutants (**Figure 5A**), whereas in the Jemalong A17 background, the petiolules of lateral leaflets appear to come from the same location on the petiole (Chen et al., 2010).

Scanning electron microscopy analysis shows that all five leaflets of pentafoliate leaves in the *mtphan;mtago7* double mutant were subtended by the motor organ, pulvinus, similarly as in WT (R108), and *mtphan* and *mtago7* mutants (**Figures 5B–J**). In addition, the two lateral leaflets had elongated petiolules, resembling the phenotype of the *palm1* mutant, particularly in the R108 background (**Figures 5A,E,H**) (Chen et al., 2010). Consistent with the leaf patterning phenotype, *PALM1* expression was significantly downregulated in the vegetative shoot buds in the *mtphan;mtago7* double mutants (**Figure 5L**). These results suggest that transcriptional and post-transcriptional mechanisms act synergistically in restricting the expression of *MtARF3*, which, in turn; negatively regulate the expression of *PALM1*.

**FIGURE 6:**
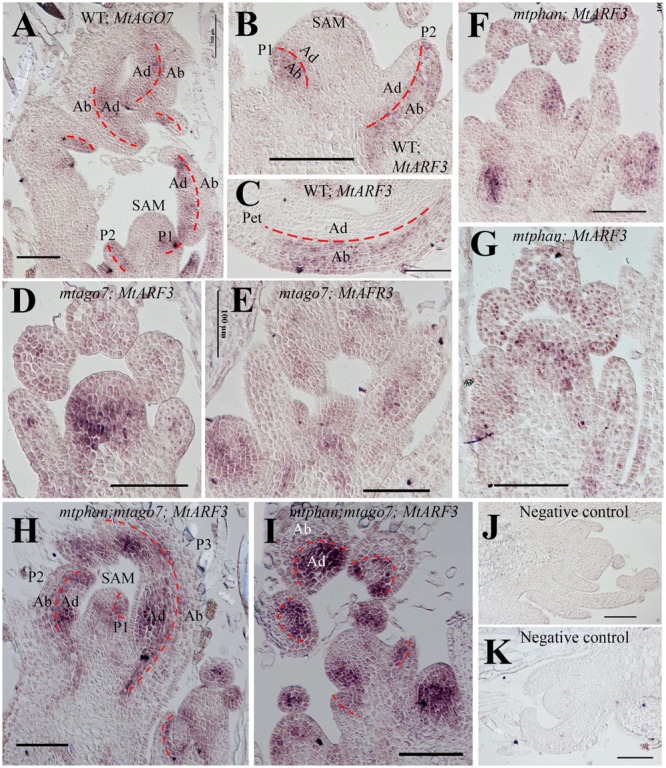
*In situ* hybridization analyses of *MtARF3* and *MtAGO7* gene expression in *M. truncatula*. **(A)** Localization of *MtAGO7* transcripts in vegetative shoot buds in WT. **(B,C)** Localization of *MtARF3* in vegetative shoot bud **(B)** and petiole of a developing leaf **(C)** in WT. Note that in wild type plants, *MtAGO7* and *MtARF3* expressed in opposite domains in leaf primordia, with *MtAGO7* being expressed in the leaf adaxial domain and *MtARF3* in the leaf abaxial domain. **(D,E)** Localization of *MtARF3* gene expression in *mtago7* mutant. *MtARF3* gene expression was detected in both abaxial and adaxial domains of leaf primordia in the *mtago7* mutant. **(F,G)** Localization of *MtARF3* gene expression in *mtphan* mutant. *MtARF3* gene expression was detected in both abaxial and adaxial domain of leaf primordia in the *mtphan* mutant. **(H,I)** Localization of *MtARF3* gene expression in *mtphan;mtago7* double mutant. *MtARF3* gene expression was detected in both abaxial and adaxial domains of leaf primordia in the *mtphan;mtago7* double mutant, with stronger signals in the adaxial domain. **(J,K)** Negative controls using sense probes in neighboring sections for detection of *MtAGO7* **(J)** and *MtARF3* **(K)**. Scale bars: 100 μm. Dashed lines mark the boundary between the adaxial and abaxial domains. SAM, shoot apical meristem; P, plastochron; Ab, abaxial domain; Ab, abaxial domain.

RNA *in situ* hybridization results show that in the vegetative shoot apex in wild type, *MtARF3* transcripts were detected in the SAM and the abaxial domain of leaf primordia (**Figures 6B,C**). Its expression domain is opposite from *MtAGO7* (**Figure 6A**) (Hunter et al., 2006; Iwasaki et al., 2013; Zhou et al., 2013; Ge et al., 2014). In *mtphan* and *mtago7* mutants, *MtARF3* transcripts were detected in the adaxial domain as well as the abaxial domain (**Figures 6D–G**), consistent with the notion that both AGO7 (*tasiR-ARF* pathway) and PHAN restrict the *ARF3* expression in the abaxial domain. Similarly, in the *mtphan;mtago7* double mutants, *MtARF3* transcripts were detected in both adaxial and abaxial domains of developing leaf primordia; and *MtARF3* transcripts appeared much higher in the adaxial domain than the abaxial domain and corresponding domains in wild type, and *mtphan* and *mtago7* single mutants (**Figure 6**).

### Ectopic Lateral Leaflets Are Initiated at a Late Developmental Stage Similarly As in the *palm1* Mutants

**FIGURE 7:**
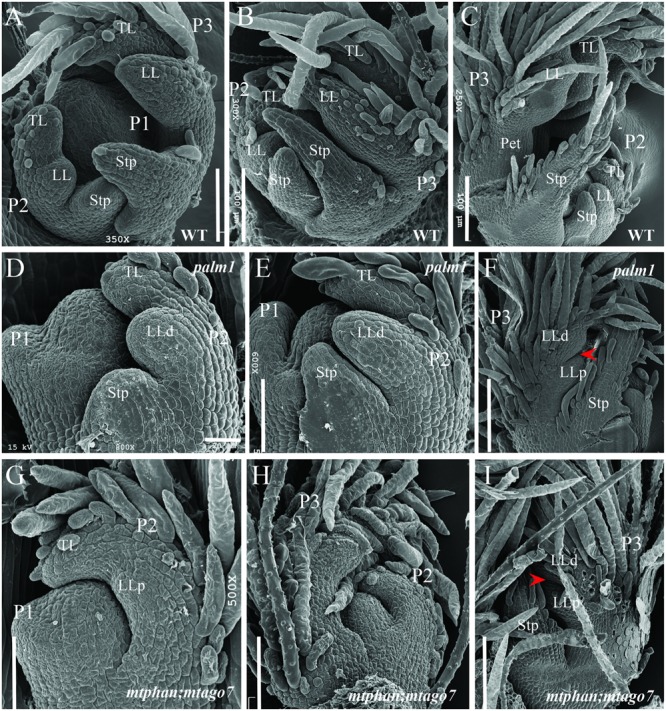
Scanning electron microscopic analyses of leaf primordia development in WT, and *palm1* and *mtphan;mtago7* mutants. SEM images of leaf development in WT **(A–C)**, *palm1* **(D–F)**, and *mtphan;mtago7* **(G–I)**. Some trichomes were removed in order to view the boundary between LLd and LLp **(I)**. Arrowheads **(F,I)** denote the boundary between LLd and LLp. P, plastochron; TL, terminal leaflet; LL, lateral leaflet; Stp, stipule; LLd, distal lateral leaflet; LLp, proximal lateral leaflet.

In the *palm1* mutant, leaf developmental process is indistinguishable from wild type until the P3 stage, when an additional pair of lateral leaflet primordia (LLp) starts to initiate at the base of the lateral leaflet primordia (LLd) (**Figures 7A–F**) (Wang et al., 2008; Chen et al., 2010). We investigated the timing of initiation of the ectopic pair of lateral leaflets in the *mtphan;mtago7* double mutant. SEM analysis shows that the common leaf primordia initiated normally in the *mtphan;mtago7* double mutant as in wild type (**Figure 7G**) (Wang et al., 2008; Chen et al., 2010). However, subsequently, trichomes developed precociously before lateral leaflet and stipule primordia initiate. At the P2 stage, lateral leaflet primordia (LL) initiated at the marginal blastozone of the common leaf primordia (**Figures 7G,H**). And, lateral leaflets expanded and trichomes covered all of the terminal and lateral leaflet primordia (**Figure 7H**). Subsequently, at the P3 stage, an additional pair of lateral leaflet primordia (LLp) initiated at the base of the distal lateral leaflet primordia (LLd; **Figure 7I**). Although LLp initiated at similar position and time as in the *palm1* mutant, they were narrower than those formed in the *palm1* mutant starting from the P2 stage. In addition, stipule primordia were sometimes not initiated in the *mtphan;mtago7* mutant. These results suggest that the initiation of ectopic lateral leaflets was similar between the *mtphan;mtago7* double mutant and the *palm1* mutant.

### Leaves of the *mtphan;mtago7* Double Mutant Were Abaxialized

Both PHAN and AGO7 regulate leaf adaxial-abaxial polarity (Xu et al., 2006; Iwasaki et al., 2013). In *A. majus*, loss of function *phan* mutant develops abaxialized needle-like leaves (Waites and Hudson, 1995). In *crispa*, a *phan* mutant in pea (*Pisum sativum*), compound leaves remain pinnate, with individual leaflets abaxialized (Tattersall et al., 2005). It has been shown that the *mtphan* mutant displays a mild polarity defect at a late developmental stage (Ge et al., 2014). SEM observation shows that the epidermal cells of the *mtphan* mutant is similar as wild type, with abaxial epidermal cells exhibiting the jigsaw puzzle-shaped border and adaxial epidermal cells exhibiting smoother border (Supplementary Figures S3A–C,F,G) (Ge et al., 2014). In the *mtago7* mutant, both the adaxial and abaxial epidermal cells are slightly elongated compared with wild type, suggesting that the adaxial domain is abaxialized in the *mtago7* mutant (Supplementary Figures S3A,B,D,F,H). In the *mtphan;mtago7* double mutant, epidermal cells on the adaxial surface were slightly elongated, similar as those of the *mtago7* mutant (Supplementary Figures S3A,B,D,E); epidermal cells in the proximal region on the abaxial surface were elongated with smooth borders, which are reminiscent of epidermal cells of midveins (Supplementary Figures S3I,J). Epidermal cells at the distal margin are radialized with elongated and smooth cells (Supplementary Figures S3K–M). These results are consistent with a role of *PHAN* and *tasiR-ARF* pathways in leaf adaxial-abaixal polarity regulation in plants.

### *SGL1* and *NAM* Are Required for Lateral Leaflet Development in *mtphan;mtago7* Double Mutant

**FIGURE 8:**
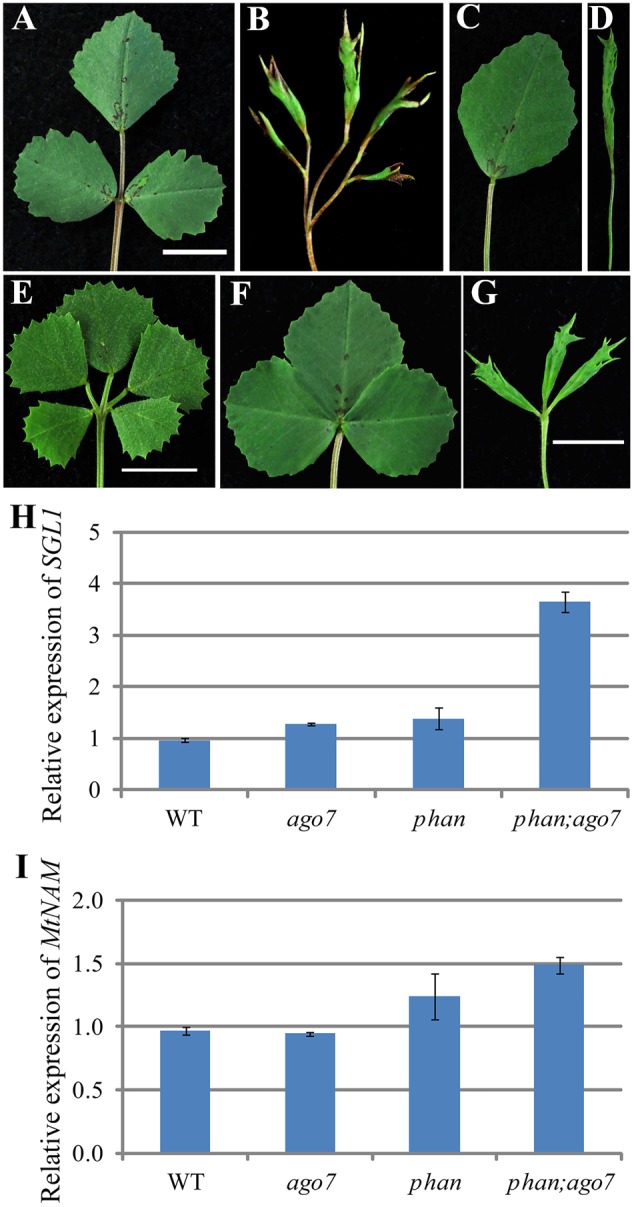
*SGL1* and *NAM* are required for the development of ectopic lateral leaflets in the *mtphan;mtago7* double mutant. Images of mature leaves in WT **(A)**, *mtphan;mtago7* **(B)**, *sgl1* **(C)**, *mtphan;mtago7;sgl1* **(D)**, *palm1* **(E)**, *mtnam* **(F)**, and *mtphan;mtago7;mtnam* **(G)**. **(E)** Was the same as in **Figure 5A**. The *mtphan;mtago7;sgl1* triple mutant develops simple leaves similarly as *sgl1*, and the *mtphan;mtago7;mtnam* triple mutant develops trifoliate leaves with greatly reduced rachis, resembling leaf phenotype of the *mtnam* mutant. Quantitative RT-PCR analyses of transcript levels of *SGL1* **(H)** and *MtNAM* **(I)** in WT, and *mtago7, mtphan*, and *mtphan;mtago7* mutants. Shown are means ± SD; *n* = 3.

The *Medicago LFY/UNI* ortholog *SGL1* is required for lateral leaflet initiation in *M. truncatula* (Wang et al., 2008; Chen et al., 2010; Peng et al., 2011). To test genetically the requirement of *SGL*1 in the development of pentafoliate leaves in the *mtphan;mtago7* double mutant, we generated *sgl1;mtphan;mtago7* triple mutant. Phenotypic analysis shows that *sgl1;mtphan;mtago7* triple mutant plants developed simple leaves with narrow lamina (**Figures 8A–D**). Thus, the genetic interaction results confirm that the pentafoliate leaf phenotype of the *mtphan;mtago7* double mutant is dependent on *SGL1*. Thus, the epistatic interaction between *sgl1* and *mtphan;mtago7* is similar to that between *sgl1* and *palm1* (Chen et al., 2010).

In *M. truncatula, NO APICAL MERISTEM* (*NAM*)/*CUP-SHAPED COTYLEDON2* (*CUC2*) has been shown to regulate organ boundary establishment (Cheng et al., 2012). Our phenotypic analysis shows that *mtnam;palm1* double mutant exhibited primarily the phenotype of the *mtnam/cuc2* mutant, indicating that *nam/cuc2* is epistatic to *palm1* (Supplementary Figure S4) To further test genetic interactions, we also generated *mtnam;mtphan;mtago7* triple mutant. Phenotypic analysis shows that the *mtnam;mtphan;mtago7* triple mutant developed trifoliate leaves with greatly reduced rachis, resembling the *mtnam/cuc2* mutant phenotype (**Figures 8E–G**). The epistatic interaction shows that *MtNAM/CUC2* is required for the elaboration of palmate-like pentafoliate leaves in the *mtphan;mtago7* double mutant, similar to that in the *palm1* mutant (**Figures 8E–G** and Supplementary Figure S4). Consistent with the phenotypic analyses, the transcript level of *SGL1* is slightly increased in *mtago7* and *mtphan* single mutants and greatly increased in the *mtphan;mtago7* double mutant (**Figure 8H**), and the transcript level of *MtNAM/CUC2* was significantly increased in the *mtphan* mutant and even more so in the *mtphan;mtago7* double mutant (**Figure 8I**).

## Discussion

In both simple and compound leaf species, initiation of leaf primordia requires downregulation of the class I knotted-like homeodomain proteins (KNOXI) in incipient leaf primordia. In compound leaf species, including tomato and *C. hirsuta*, the *KNOXI* genes are reactivated in developing leaf primordia and this reactivation is required for compound leaf development in these species. In some legume species including pea and *M. truncatula*, the KNOXI genes are not reactivated in developing leaf primordia and thus not likely involved in the development of compound leaves. Instead, UNI in pea and SGL1 in *M. truncatula* act in place of KNOXI in mediating the initiation of leaflet primordia in these legume species. In both simple and compound leaf species, it has also been shown that local gradients of auxin (auxin maxima) mediated by the auxin transport PIN-FORMED (PIN) proteins are required for the initiation and positioning of leaf primordia along the periphery of the SAM (Koenig et al., 2009; Peng and Chen, 2011; Vernoux et al., 2011). In compound leaf species, auxin maxima are also required for development of leaf margin serrations and initiation of leaflets (Peng and Chen, 2011; Ben-Gera et al., 2012). However, the underlying molecular mechanisms remain largely elusive.

Auxin signaling is dependent on AUXIN RESPONSE FACTORs (ARFs), which activate or repress downstream gene expression, and Aux/IAA transcription repressors that interact with ARF proteins. At a high auxin level, Aux/IAA proteins are ubiquitinated and degraded by the SCF^TIR1/AFBs^ 26S proteasomes, and thus releasing their inhibitory effects on ARF’s transcriptional activities (Dharmasiri et al., 2005; Kepinski and Leyser, 2005). In tomato that forms dissected leaves, the IAA9 protein, ENTIRE (E) accumulates between initiating leaflet primordia and is required for leaflet separation, since the loss-of-function *e* mutant exhibits simplified leaves compared with dissected leaves in wild type plants (Ben-Gera et al., 2012). In *M. truncatula* that forms trifoliate leaves, a loss-of-function mutation of *PIN10*, encoding an auxin efflux protein, results in altered positioning and fusion of common leaf primordia at the periphery of SAM and smooth leaflet margins (Peng and Chen, 2011; Zhou et al., 2011).

It has been shown that loss-of-function *sgl1* or *uni* mutants develop simplified leaves (Hofer et al., 1997; Champagne et al., 2007; Wang et al., 2008). On the other hand, loss-of-function mutations in *PALM1* result in dissected leaves with five leaflets in contrast to dissected leaves with three leaflets in wild type plants (Chen et al., 2010; Ge et al., 2010). PALM1 has been shown to directly interact with the *SGL1* promoter sequence and negatively regulate transcription of *SGL1*, thus serving as a negative regulator of dissected leaf development in *M. truncatula* (Chen et al., 2010; Ge et al., 2010).

Here, we show that *PALM1* gene expression is negatively regulated by AUXIN RESPONSE FACTOR3 (ARF3) in *M. truncatula*. Our bioinformatics analysis shows that there are 18 TGTCXX *cis*-elements, resembling the canonical auxin response element (AuxRE; TGTCTC) in the 2.5 kb promoter region of the *PALM1* gene. We show that in the presence of three tandem repeats of several TGTCXX elements, ARF3 significantly repressed reporter gene expression in the transient transcription activity assay in tobacco. We noticed that there are different background activities when different elements were assayed in tobacco. We speculated that this may be due to the activity of endogenous ARF3 proteins in tobacco cells. By comparing the reporter gene expression in the presence of ARF3 and the unrelated β-glucuronidase (GUS) protein, we reasoned that any influence of background activity on the assay should be minimized. In *M. truncatula*, chromatin immunoprecipitation (ChIP) assay shows that P1, P2 and P4 regions, which harbor the TGTCXX elements, but not P3 region, which does not contain any TGTCXX elements, of the *PALM1* promoter sequence were enriched in ARF3-bound chromatin. The P2 region of the *PALM1* promoter sequence contain four TGTCXX elements. However, in the transcription activity assay in tobacco, three tandem repeats of individual elements did not confer ARF3 inhibitory effects on reporter gene expression. There may be several possibilities to account for the discrepancy between the two assay results: (1) three tandem repeats of the same element may not be optimal for the binding of ARF3 in tobacco cells; (2) the spacing between tandem repeats may not be optimal for ARF3 binding in tobacco; and (3) the heterologous system (tobacco cells) may not be optimal for ARF3 binding of the TGTCXX elements. Nevertheless, the combined results strongly support that *M. truncatula* ARF3 interacts with the TGTCXX *cis*-elements in the *PALM1* promoter sequence *in vivo*.

**FIGURE 9:**
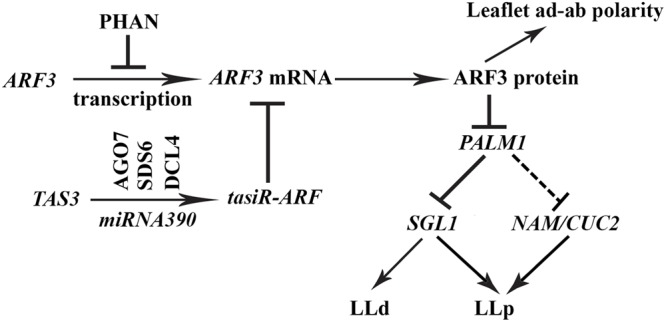
A genetic model illustrating the role of the ARF3-PALM1 module in dissected leaf development in *M. truncatula*. The MYB domain protein PHAN negatively regulates transcription of *ARF3* and the *trans*-acting short interfering RNA, *tasiR*-ARF, whose biogenesis is dependent on the AGO7-SDS6-DCL4-miRNA390 pathway, negatively regulates the transcript level of *ARF3*. ARF3 in turn negatively regulate the expression of *PALM1*, a negative regulator of the *Medicago LEAFY* ortholog, *SGL1*, which is required for the initiation of lateral leaflets in *M. truncatula*. A fine-tuned expression of *PALM1* and *SGL1* is crucial for the development of trifoliate leaves in *M. truncatula*. *NAM/CUC2*, whose expression is repressed directly or indirectly by *PALM1*, is required for the initiation of proximal lateral leaflets (LLp) in the *palm1* mutant. Solid and dashed lines denote confirmed and unconfirmed molecular interactions, respectively. LLd, distal lateral leaflets.

Consistent with previous reports, the MYB domain protein, PHANTASTICA (PHAN), negatively regulates the expression of *AUXIN RESPONSE FACTOR3* (*ARF3*) in *Medicago* plants and the *trans*-acting short interfering RNA, *tasiR-ARF* pathway negatively regulates the transcript level of *ARF3* (**Figure 9**) (Iwasaki et al., 2013). Both *PHAN* and *tasiR*-*ARF* pathways mediate adaxial-abaxial polarity in diverse species. When both pathways are compromised as in the *mtphan;mtago7* double mutant, the dissected leaf patterning changes from trifoliate to palmate-like pentafoliate leaves (**Figure 5**), in addition to narrow blades. The change in the dissected leaf patterning is associated with a drastically increased level of *ARF3*, decreased level of *PALM1* (**Figure 5**) and a greatly increased level of *SGL1* (**Figures 8H, 9**). Independent evidence that supports this conclusion includes (1) when the *tasiR*-ARF resistant *ARF3^m^* is highly overexpressed, dissected leaf patterning changes from trifoliate to palmate-like pentafoliate leaves, albeit with radialized blades (**Figure 4**); (2) *sgl1;mtphan;mtago7* triple mutant exhibits simple leaves with narrow blades, similar to *sgl1;palm1* double mutant (**Figure 8**) (Chen et al., 2010); and (3) *nam;mtphan;mtago7* triple mutant exhibits trifoliate leaves with greatly reduced rachis, indicating that *NAM* is required for the development of ectopic lateral leaflets in the *mtphan;mtago7* double mutant as in the *nam;palm1* mutant (**Figure 8** and Supplementary Figure S4).

The *PALM1-SGL1* module is important for the development of trifoliate leaves in *M. truncatula*. Our results that the PHAN and *tasi*-ARF pathways, which are known adaxial-abaxial regulators, are recruited to fine tune the expression level and domain of *PALM1* and therefore regulate the compound leaf patterning in *M. truncatula* (**Figure 9**) are consistent with the observation that auxin and AS1 converge at the incipient leaf primordia to control leaf development in *A. thaliana* that has simple leaves (Hay et al., 2006). In conclusion, the multilayered regulation of gene expression suggests that a properly balanced expression of key regulators including *PALM1, SGL1* and likely *NAM/CUC2* is critical for the development of trifoliate leaves in the legume plant, and this process requires auxin signaling (**Figure 9**).

## Author Contributions

JP and RC: designed experiments; JP and AB: performed the experiments; JP, FM, and RC: analyzed data; JP and RC: wrote the paper.

## Conflict of Interest Statement

The authors declare that the research was conducted in the absence of any commercial or financial relationships that could be construed as a potential conflict of interest.

## Footnotes

1 http://medicago.jcvi.org/medicago/

2 http://www.megasoftware.net/
