# Supplementary material for: AUXIN RESPONSE FACTOR3 Regulates Compound Leaf Patterning by Directly Repressing PALMATE-LIKE PENTAFOLIATA1 Expression in Medicago truncatula
Source: Front Plant Sci. 2017 Sep 20;8:1630. doi: 10.3389/fpls.2017.01630 (PMC5611443; doi:10.3389/fpls.2017.01630)
Supplement: Supplementary file 1 [file Data_Sheet_1.PDF]

Supplementary Material

AUXIN RESPONSE FACTOR3 regulates compound leaf patterning by directly repressing *PALMATE-LIKE PENTAFOLIATA1* expression in *Medicago truncatula*

Jianling Peng[1], Ana Berbel[2], Francisco Madueño[2] and Rujin Chen[1,*]

[1]Noble Research Institute, 2510 Sam Noble Parkway, Ardmore, Oklahoma 73401; [2]Insituto de Biología Molecular Celular de Plantas (IBMCP) , Consejo Superior de Investigaciones Científicas, Universidad Politécnica de Valencia, CPI, Ed. 8E, Ingeniero Fausto Elio s/n, 46011 Valencia, Spain.

Supplementary Fig. S1. Putative auxin response elements (AuxREs; TGTCXX) in the *PALM1* promoter sequence. (**A**) Distribution of putative AuxREs in the 2.5-kb *PALM1* promoter sequence. Putative AuxREs located in the "+" and "-" strands of the promoter sequence are highlighted in yellow and green, respectively. The promoter sequence is divided into four partially overlapping subfragments (from top to bottom: P1, letters underlined; P2, letters in red; P3, letters underlined; and P4, letters in blue). (**B**) Sequences and locations of 10 different putative AuxREs in the 2.5-kb *PALM1* promoter sequence.

Supplementary Fig. S2. Phylogenetic analysis of AUXIN RESPONSE FACTORs (ARFs) in *M. truncatula* and *A. thaliana*. A maximum likelihood phylogenetic tree was reconstructed using MEGA10.

Supplementary Fig. S3. Phenotypic analysis of leaf phenotypes. (**A**) Abaxial views of mature leaves in WT (R108), and *mtphan*, *mtago7* and *mtphan;mtago7* mutants. (**B-E**) Morphology of leaf adaxial epidermal cells of R108 (**B**), *mtphan* (**C**), *mtago7* (**D**) and *mtphan;mtago7* (**E**). (**F-I)** Morphology of leaf abaxial epidermal cells of R108 (**F**), *mtphan* (**G**), *mtago7* (**H**) and *mtphan;mtago7* (**I**). Images were taken at the areas shown in (**A**). (**I**) Abaxial surface epidermal cells in *mtphan;mtago7* mutant. (**J**) Abaxial view of the proximal region of a leaflet in the *mtphan;mtago7* mutant. A close-up view of the boxed area in (**J**) was shown in (**I**). (**K**) Adaxial view of the distal region of a leaflet in the *mtphan;mtago7* mutant. Arrowheads show abaxialized serration tips. (**L**) A close-up view of an abaxialized serration tip marked in (**K**). (**M**) A P5 stage leaf of the *mtphan;mtago7* mutant, showing that an ectopic lateral leaflet (arrowhead) initiated from the adaxial domain of a developing lateral leaflet and all leaflet blades failed to expand.

Supplementary Fig. S4. Genetic interaction between *mtnam1* and *palm1*. (**A**) Representative images of mature leaves of R108, *nam*, *palm1* and *nam;palm1*. (**B**) Four different types of leaves were observed in the *nam;palm1* double mutant. These include (1) three leaflets clustered together; (2) three leaflets fused together, but with three separate midveins; (3) four leaflets fused together; and (4) five leaflets fused together. (**C**). Percentage of four different types of leaves in the *nam* and *nam;palm1* mutants.

Supplementary Table S1. Primers used in this study.

A

ATATCTAATTTTTTAATTATTTAATGAATAAAAACAACAAGCTTAACATGTTTAGCCCGCAAGTGAAGTATAC
TTAATTAACTAATACTAGGTATGAGTTAGCTAGCATCCCTGTCTGTGAATTTGGTGGCATGTACTAGTTGGA
CGTCGACATAAACAATGATACAGGAACGTAAGAAAGGTGTGTTTCTTTCTTTCTTGGTATGTTCGTATATAT
ATATATATAGGCGAAGATAGCTATGTTTTCAAAGTAGCTATATGTTACTTCATCAGCTATCTTTTGTTAATTG
TCTTATATGGATTCAATCAATTAATTAATTGTCCAACGGGTTTTATAATTTGAATGCGCGTAATGAGAGTTTT
ATTTGTCTCTGCAGTTGCAGGGGGGCTATGCATACTGACAACAAGAAACTAACAGAATAGAGCATTAGAGA
CCTACCTAGCTTTGCCTTCTACCATTCTTTCAACATGATAATATAATTATTTTTTTTTTTTTTTGTAAGATCT
CAACAATAACAATAGGTACAATGCAAGCAACTTAATCACTGATCATAAAAAAATATTTTAAAGGCCGTCAAG
TATTTTGGAAACAAATATTTTATTAAAAGATTTTACTAACCATTATCCTTAGGGTAATTGTTAAGGAGTTCAA
AAATAGAAAATTTTCATTAAAAACAACAACCTTTTAACTTTCATAAAGTTAAATATTCTACTTTTCAACAATT
TCGTCTAATGCAGAATTTTTATATTTATCTTTTTAACCAACTCCCTTAGGGCACTGATTAACATTTTTCTTTAT
TAAATAATTGACGTATCAAAATTATAACATGGTTTAGATACATCAATTATTTAATAAATCTAAAAAAAAAAAA
ATTTTTGTTTAAAACAGTGACTTGAAGAAATATATATGTATAAATATTGCTTCTAATTTGTTTTTTTCTTTTAA
AAAGGCCAAAAGAATATGATATAGGAAAATCGGAGTAGAAGACATACTTTAAATCAAATGATGGTAGGTGT
TTATAGGTTATGGTCTTTGCAGTGGTGATTTTTTTCGGATGCCATAGAATTTCTTCTTCACATGTGTATTTTA
TTGTATCAGTGGCTGACATTGGATTTTCTTCCTATTGTCTGATGTTGAGGTTACTTTGTACATCTTGTGATTA
TTGGCTCTTTATCAATAAAAACATGGAAAGAACATAAAAGACAACAAAAACAAATAATTTAAATGAGATGGT
TCACATATTTTTTAGTTTAATAAGAGTTTAAGAATTTGATAGAAACGGAATTGCAAAGCCCATAACTCTCTTT
TCGTAGCATTTAGAACTTTATATTTGTAAAATGATTTCAAACAACTAGTATATATTGAATTAACGTGTGATGG
TTTTACATTTATTAACTCAGAAATTCTAATGAAAATAGGTAATGGAGTATTTATATTTGGGAAATTTTCATAT
TAAAAAAATAGGGAGTGTAATAATTTTTTTTTTTTTTTTTGAGGGGAAGGAGTGTAATAAATTTTAATCAAA
TAGTACCTTTTATCCATTATTTTTGTGAATATCAAATTTATTTTTTTACTAAAAATGATAAGGATCCATCTTTG
AACTATTTTTTAGAGGTCCACACATTAGATGCACCCTTAAAAAAATTATATTAGCATATGAATAAAGTAATAA
ACTAATTTATGAATTAATGTTATTCGTATTATGTTATTTTAAGAGGTAGAACAAGTTGGGTCAAACAATATTT
TTTTTAAAGATTTTGATTACTATATATCTTAGAAGCATTTTAATGAAATATTTATTATTTTAAAATTCATTTTT
ATTAAAAATAGTAAATGTTTATATTTGGAAGAATTTTGAATGATTCATATTAGATTGACCCTATATAGGCAGT
TACTTTTATGTATTTCTACTTTTTTTAGGAGATGTATATTTATTTTGTATAGCCTTTTTACATTTTGAATGCTT
AATGTCAAAATTAAGGACATATTAAACTTATATTGCGATTTGCAACTAATTAAAAATTAAAAAGAGGGTGAG
GAATATTGAAGATTTAAAACAATGGTGTGCTTTCCCCCTCAAAAAATAATTGTGTGCTTGATGAGTTATATTT
CAATGACGTAGATGTCAAAGGAGATGATTTGTCTATATTAAGCAATAGCTAGTTGTATGTGCTTCAATTATA
ACCTATAGTATTTTTTTATTTACCATTATTAGTATAATATATCCTTTTTTTTTTTTGAGGGAGTAGTATAGTAT
AATATATCTAGTGTCAATATGTTCCAATCCAGCAGCTATCTATCTAGCTTACTTAAAATTTATTAATTGGTGA
TACATTCGATTTGATGTTTTAAAGCATGACTTTGTCTTGACCTTAAATATTATTAATTATATTCACCCCCCCT
ATTTAATAACGAATTATATTTATCTCCAATTTCATCCCCCACCCCATTACCAATATCTATCTATCTTTCTATCT
TAAATGTCAGTGACAGTACC

B

Name	Sequence	Strand	Site	Position in the promoter region		
E1	TGTCTC	+	1	(-2134, -2129)		
E2	TGTCAA	+	3	(-529, -524)	(-374, -3690	(-229, -224)
E3	TGTCAC	-	1	(-11, -6)		
E4	TGTCAG	+	1	(-16, -11)		
		-	2	(-1392, -1397)	(-2098, -2103)	
E5	TGTCCA	+	1	(-2176, -2181)		
E6	TGTCCT	-	1	(-513, -518)		
E7	TGTCGA	-	1	(-2353, -2348)		
E8	TGTCTA	+	1	(-357, -352)		
E9	TGTCTG	+	2	(-1374, -1369)	(-2383, -2488)	
E10	TGTCTT	+	2	(-135, -130)	(-2207, -2212)	
		-	2	(-1294, -1299)	(-1510, -1515)	

Supplementary Fig. S1.

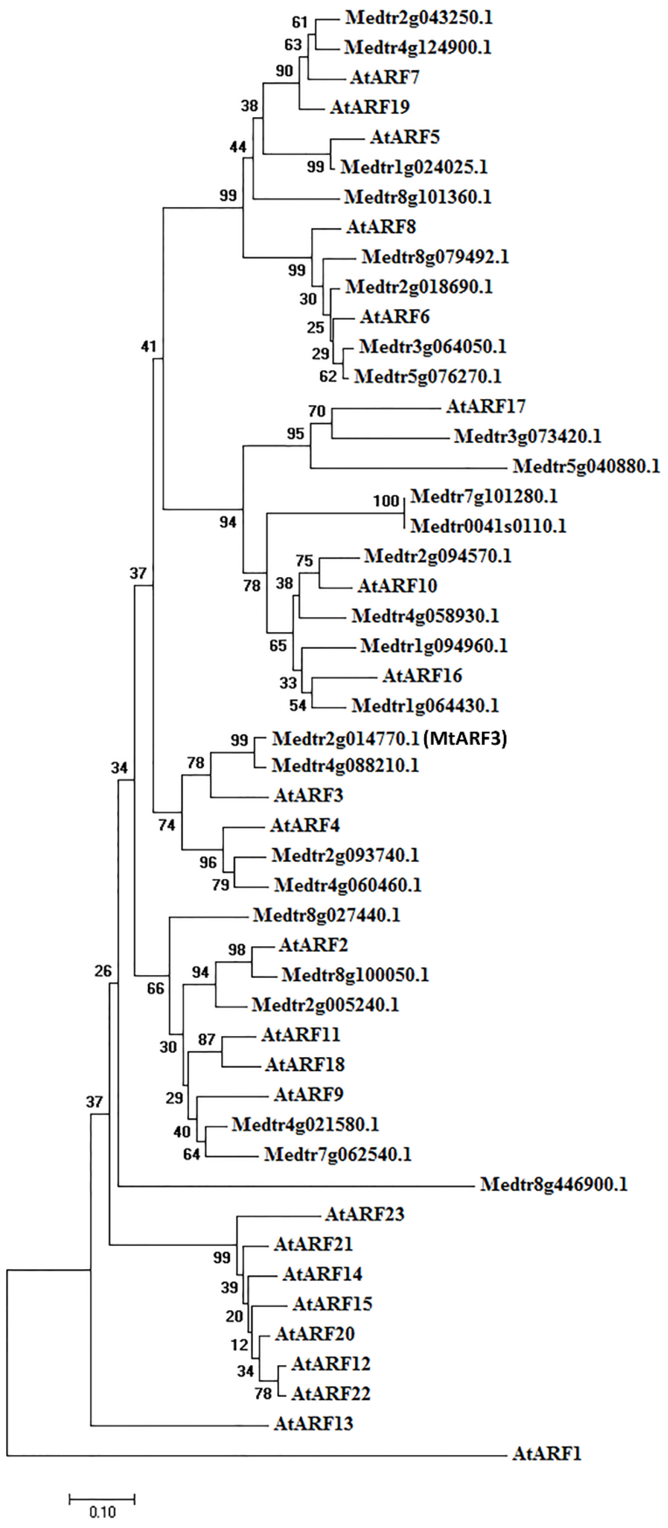

Supplementary Fig. S2.

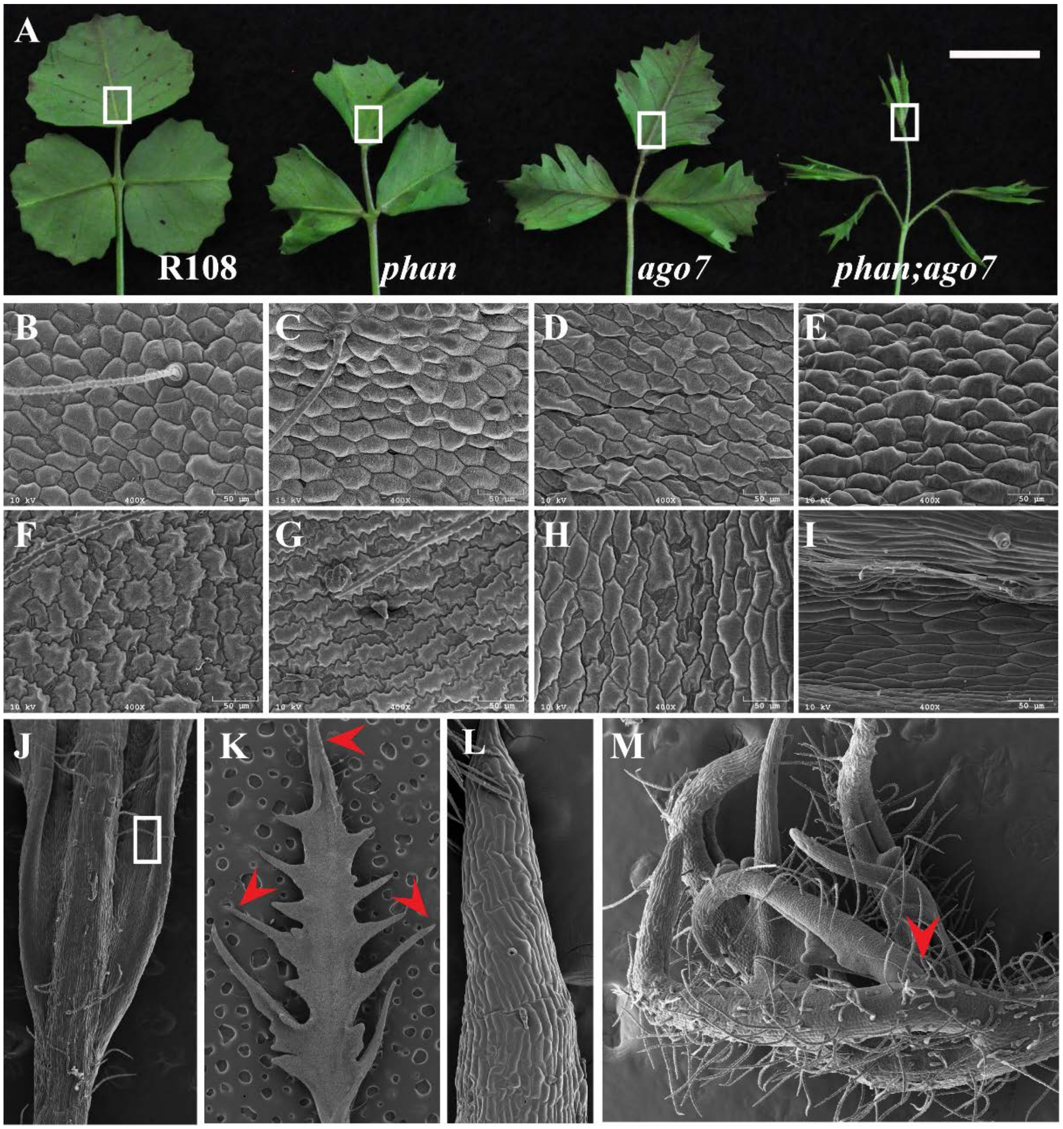

Supplementary Fig. S3.

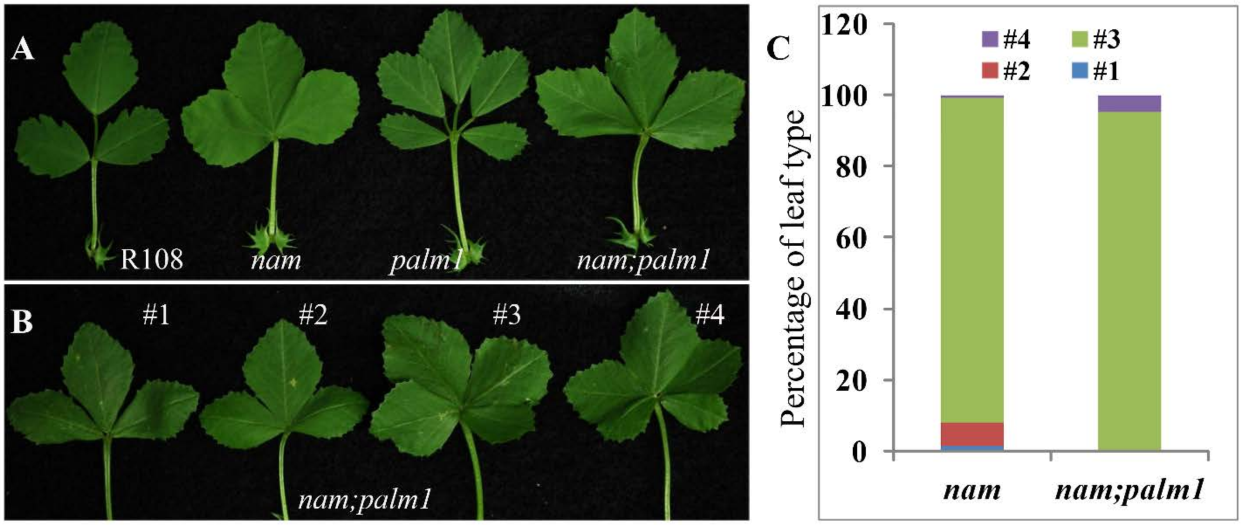

Supplementary Fig. S4.

Supplementary Table S1. Primers used in this study		
**Primer**	**Sequence**	**Purpose**
MtARF3-F	CACCATGGGGTTAATCGATCTCAACAC	MtARF3
MtARF3-R	TCAACCGCCGCCAATGTGTGTG	MtARF3
$MtARF3^m$-FA	CGAAAAGTACTTCAGGGACAGGAGATTATGGGCATGAATACTCC	$MtARF3^m$
$MtARF3^m$-RA	AATCTCCTGTCCCTGAAGTACTTTTCGGAATCTTAATGATTCCCCAAAG	$MtARF3^m$
$MtARF3^m$-FB	CAAAAAGTACTTCAGGGACAGGAGATTCTTCTGTGCCCACCCTAC	$MtARF3^m$
$MtARF3^m$-RB	AATCTCCTGTCCCTGAAGTACTTTTTGGAATCTGAAAGATTCACC	$MtARF3^m$
$MtARF3^m$-MID-F	AAAGTACTTCAGGGACAGGAGATTATGGGCATGAATACTC	$MtARF3^m$
$MtARF3^m$-MID-R	CTCCTGTCCCTGAAGTACTTTTTGGAATCTGAAAGATTC	$MtARF3^m$
PALM1-P1-F	ATCGGTACCGAGTTAGCTAGCATCCCTGTC	ChIP-PCR
PALM1-P1-R	ATGCTCGAGTTCTGCATTAGACGAAATTGTTG	ChIP-PCR
PALM1-P2-F	ATCGGTACCTTAGGGTAATTGTTAAGGAGTTC	ChIP-PCR
PALM1-P2-R	ATGCTCGAGTTCTAAATGCTACGAAAAGAGAG	ChIP-PCR
PALM1-P3-F	ATCGGTACCGAGATGGTTCACATATTTTTTAG	ChIP-PCR
PALM1-P3-R	ATGCTCGAGCATCTCCTAAAAAAAGTAGAAATAC	ChIP-PCR
PALM1-P4-F	ATCGGTACCAGTAAATGTTTATATTTGGAAGA	ChIP-PCR
PALM1-P4-R	ATGCTCGAGGGTACTGTCACTGACATTTAAGA	ChIP-PCR
35S -F1	ATCGGATCCGTCACGACGTTGTAAAACG	35S
35S-R1	ATGCCATGGGGGATCCTCTAGAGTCGAGG	35S
35S-E2-R	AAGGTTGACAAAAGGTTGACAAAAGGACTAGTGAAGGATAGTGGGATTGTGC	35S-E2
35S-E2-F	AACCTTTTGTCAACCTTTTGTCAAGCGGCCGCGCAAGACCCTTCCTCTATATAAG	35S-E2
E1-F	ACTAGTCCTTTTGTCTCCCTTTTGTCTCCCTTTTGTCTCGCGGCCGC	E1
E1-R	GCGGCCGCGAGACAAAAGGGAGACAAAAGGGAGACAAAAGGACTAGT	E1
MtARF3-RT-F	GAGCCATCTGGTTCTGCTTC	RT-PCR
MtARF3-RT-R	GAAGTTGTGCCTTCCAGAGC	RT-PCR
MtPHAN-RT-F	GCAACACACGGAAACAAATG	RT-PCR
MtPHAN-RT-R	GGTAAGGCGTAAATGCTTCG	RT-PCR
PALM1-RT-F	CACCATGGCTACAAGATATTGGCC	RT-PCR
PALM1-RT-R	TCAAGTTGGTGTTGGCTTGTTC	RT-PCR
SGL1-RT-F	CACCTGGATCCCGACGCATTCAC	RT-PCR
SGL1-RT-R	TTAAAAAGGAAGGTGAGCAGTTC	RT-PCR
MtAGO7-RT-F	ATGGAACAACAAGAAAACTCAACC	RT-PCR
MtAGO7-RT-R	CTAGCAGTAAAACATAAGCTTCTTGA	RT-PCR
ACTIN-RT-F	TCTTACTCTCAAGTACCCCATTGAGC	RT-PCR
ACTIN-RT-R	GTGGGAGTGCATAACCTTCATAGATT	RT-PCR
PALM1-q-F	AAACCCAACCACCGTTAAATTCT	qRT-PCR
PALM1-q-R	CACAATCCAGCATTAGCAACAAG	qRT-PCR
SGL1-q-F	GATGAACAGCCTTTCCCAGATT	qRT-PCR
SGL1-q-R	GCCGTAACGCTCTCCAACA	qRT-PCR
MtARF3-q-F	TGCACGACGCTGCTACACA	qRT-PCR
MtARF3-q-R	AGACGACGGAGACGAAACTTGT	qRT-PCR
MtARF4-q-F	GCAAGTCATGCCGATTTCGT	qRT-PCR
MtARF4-q-R	CTGGGTTCCTGATGCTTTTAGC	qRT-PCR
ACTIN-q-F	CAGTGTCTGGATCGGAGGAT	qRT-PCR
ACTIN-q-R	TGAACAATCGATGGACCTGA	qRT-PCR
PALM1-P-F	ATCGGTACCGAGTTAGCTAGCATCCCTGTC	Y1H
PALM1-P-R	ATGCTCGAGGGTACTGTCACTGACATTTAAGA	Y1H

Supplementary Table S1.
